# Hospital oral food challenge tests in the coronavirus disease 2019 pandemic: A nationwide survey

**DOI:** 10.1002/clt2.12273

**Published:** 2023-07-01

**Authors:** Noriyuki Yanagida, Chizuko Sugizaki, Sakura Sato, Motohiro Ebisawa

**Affiliations:** ^1^ Department of Allergy Clinical Research Center for Allergy and Rheumatology National Hospital Organization Sagamihara National Hospital Sagamihara Kanagawa Japan

To the Editor:

Oral food challenge (OFC) tests are necessary in food allergy practice.^1^, ^2^, ^3^, ^4^ The spread of the Omicron variant of severe acute respiratory syndrome coronavirus 2 significantly affected food allergy practice.^5^, ^6^ Universal pre‐admission screening for coronavirus disease 2019 (COVID‐19) may effectively identify patients with asymptomatic COVID‐19.^7^ However, no studies have reported the influence of the COVID‐19 pandemic and pre‐admission screening for COVID‐19 on the number of inpatient OFC tests conducted at hospitals. This multicenter nationwide cross‐sectional survey aimed to determine the number of inpatient OFC tests conducted at hospitals, infection control measures undertaken, number of nosocomial COVID‐19 infections, and positive rates for coronavirus disease 2019 (COVID‐19) following universal pre‐admission screening.

This study was approved by the Ethics Committee of the National Hospital Organization, Sagamihara National Hospital (Approval no. 2022‐013). Additional details related to the methods are presented in Supplementary Materials.

Of 373 pediatric training facilities contacted (Figure S1), 186 facilities responded to our online questionnaire (response rate, 50%), five were excluded due to missing data, and four were excluded because OFC tests had been suspended during the COVID‐19 pandemic. The data from 177 facilities were then analyzed.

Concerning OFC tests, all 177 facilities (100%) had checked body temperature, 176 (99%) had checked interview sheets, 172 (97%) had undertaken a medical check‐up prior to OFC testing, and 136 (77%) had undertaken universal masking. Concerning inpatient OFC tests, 15,858 OFC tests were conducted, including 12,503 in shared rooms and 3355 in private rooms.

Of 177 facilities, 110 (73%) had conducted universal pre‐admission screening tests for COVID‐19 for patients prior to an OFC test and 64 (37%) had conducted universal pre‐admission screening tests for COVID‐19 for both patients and family members. Of 110 facilities that conducted universal pre‐admission screening tests for COVID‐19, 55 had performed polymerase chain reaction (PCR) tests, 48 had performed rapid antigen tests, and 10 had performed nucleic acid amplification (loop‐mediated isothermal amplification [LAMP]) tests (including duplications). Samples were drawn from nasopharyngeal swabs (72%, *n* = 79) and saliva (28%, *n* = 31). The positive test rate for COVID‐19 was 0.5% (45/8219) for all patients and 0.2% (5/3172) for all family members (Table S1). Figure S2 shows the positive test rate for COVID‐19 for patients to be highest in PCR tests taken from nasopharyngeal swabs (1%, 24/2341), followed by PCR tests from saliva (0.8%, 6/773), rapid antigen tests from nasopharyngeal swabs (0.3%, 7/2211), LAMP tests from nasopharyngeal swabs (0.3%, 1/326), and LAMP tests from saliva (0.2%, 4/2254) (excluding duplication). In 20 (11%) facilities, 24 nosocomial infections in pediatric wards were reported. No nosocomial infections in wards related to OFC tests were reported.

Figure 1 presents the distribution of the number of OFC tests performed with and without universal pre‐admission screening tests for COVID‐19, with the median number being 18.5 (interquartile range [IQR], 7–80) and 56 (IQR, 18–120), respectively. Significantly fewer OFC tests were performed at Institutions that undertook universal preadmission screening tests for COVID‐19 performed significantly fewer OFC tests than those that did not undertake them (*p* = 0.001).

**FIGURE 1:**
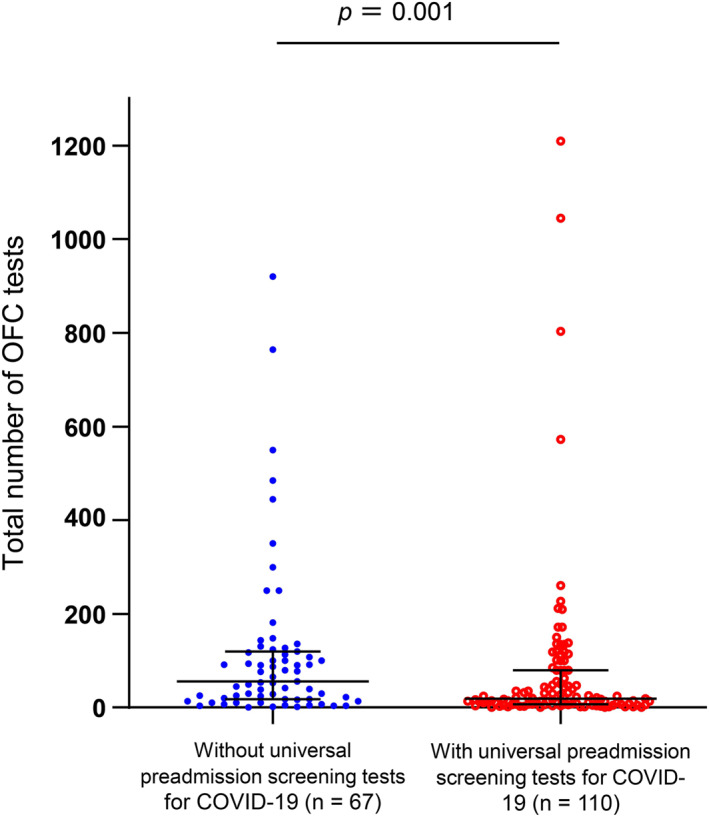
The number of Oral food challenge (OFC) tests with and without universal preadmission screening tests for COVID‐19. The median number of OFC tests for facilities with and without universal preadmission screening tests for COVID‐19 were 18.5 (7–80) and 56 (18–120), respectively. There was a significant difference in the number of OFC tests between facilities with and without COVID‐19 screening (*p* = 0.001). COVID‐19, coronavirus disease 2019; OFC, oral food challenge.

In 177 facilities, 15,858 inpatients had undergone OFC tests during the study period. Preadmission screening tests may require more staff resources and time for examinations and may therefore restrict the available number of OFC tests. The positive rate in universal pre‐admission screening tests for COVID‐19 was only 0.5%. Patients with infectious symptoms, such as fever, or those in close contact with patients with COVID‐19, may have excluded prior to hospitalization. LAMP and rapid antigen tests may be insufficient to detect asymptomatic COVID‐19 infections. One systematic review reported the impaired performance of rapid antigen tests for diagnosing COVID‐19 when the Omicron variant circulates, particularly in samples with low viral loads.^8^ In our study, all examinations, except for PCR tests, missed >75% of asymptomatic COVID‐19 infections, which might explain the low positivity rate. The COVID‐19 pandemic has affected allergy practice.^5^, ^6^ This study found that even during the COVID‐19 outbreak, many facilities were able to perform inpatient OFC tests without inducing nosocomial infections. The clinical characteristics of COVID‐19 may alter depending on the predominant variant strain at the time, and the Omicron variant may have a lower severity than other variants.^9^ Considering the low positive rate found in universal preadmission screening tests for COVID‐19 and the absence of nosocomial infection, rapid antigen and LAMP tests should not be recommended for universal preadmission screening, and OFC tests without universal preadmission screening should be considered during endemic COVID‐19.

This study had several limitations. First, 187 facilities did not respond to the survey. While most of these non‐responders may have suspended OFC tests due to the pandemic, this number of non‐responders may have resulted in selection bias. Further, Omicron is less severe than other variants.^8^ Hence, our results may not apply directly to OFC tests performed in other scenarios. Finally, the universal pre‐admission screening test could not completely exclude asymptomatic COVID‐19 infection.

In conclusion, during the COVID‐19 outbreak, many facilities safely performed inpatient OFC tests without inducing nosocomial infections. Rapid antigen and LAMP tests are insufficient to detect asymptomatic COVID‐19 infection, and universal preadmission screening tests for COVID‐19 may decrease the available number of inpatient OFC tests performed; therefore, OFC tests without a universal pre‐admission screening test should be considered during endemic COVID‐19.

## AUTHOR CONTRIBUTIONS

**Noriyuki Yanagida**: Conceptualization, Data curation, Formal analysis, Investigation, Methodology, Project administration, Validation, Visualization, Roles/Writing—original draft, Writing—review and editing. **Chizuko Sugizaki**: Conceptualization, Data curation, Investigation, Methodology, Visualization, Writing—review and editing. **Sakura Sato**: Data curation, Formal analysis, Writing—review and editing. **Motohiro Ebisawa**: Conceptualization, data curation, funding acquisition, supervision, writing—review and editing.

## CONFLICT OF INTEREST STATEMENT

Motohiro Ebisawa received lecture fees from Viatris, Sanofi and ARS‐Pharmaceuticals. The other authors have no conflicts of interest to declare.

## FUNDING INFORMATION

Health, Labour, Sciences Research Grant, Grant/Award Number: JPMH19FE1001

## Supporting information

Supporting Information S1

## Data Availability

Data supporting the findings of this study are available from the corresponding author upon reasonable request.
