# Supplementary material for: Hospital oral food challenge tests in the coronavirus disease 2019 pandemic: A nationwide survey
Source: Clin Transl Allergy. 2023 Jul 1;13(7):e12273. doi: 10.1002/clt2.12273 (PMC10314275; doi:10.1002/clt2.12273)
Supplement: Supplementary file 1 — Supporting Information S1 [file CLT2-13-e12273-s001.docx]

**Hospital oral food challenge tests in the coronavirus disease 2019 pandemic: a nationwide survey**

**Supplementary Materials**

**Supplementary Methods**

***Surveyed facilities***

This multicenter nationwide cross-sectional study concerning oral food challenge (OFC) tests included facilities affiliated with the Japan Pediatric Society that provide pediatric specialist training. There are approximately 800 pediatric specialist-training facilities and their collaborating facilities in Japan. In 2020, OFC tests were performed at 373 facilities. In Japan, 30,700 outpatient and 49,175 inpatient OFC tests are performed in pediatric training facilities annually. From July 1–31, 2022, 373 affiliated facilities were invited to complete an online questionnaire regarding the implementation of inpatient OFC tests from January 1 to June 30, 2022. Informed consent was not obtained from patients separately as this study did not include any personal information. This study was conducted in accordance with the principles of the Declaration of Helsinki and approved by the Ethics Committee of the National Hospital Organization, Sagamihara National Hospital (Approval no. 2022-013).

***Questionnaire survey***

The online questionnaire survey evaluated the number of: (i) inpatient OFC tests performed, (ii) OFC tests conducted in shared and private rooms, (iii) universal preadmission screening tests for coronavirus disease 2019 (COVID-19), and (iv) nosocomial COVID-19 infections in wards. The universal pre-admission screening test for COVID-19 included polymerase chain reaction (PCR), nucleic acid amplification (loop-mediated isothermal amplification [LAMP]), and COVID-19 rapid antigen tests from saliva or nasopharyngeal swab samples.

***Clinical outcomes***

The primary outcome was the number of inpatients who had undergone OFC tests. Secondary outcomes included the number of positive universal preadmission screening tests for COVID-19, and the number of nosocomial COVID-19 infections in wards.

***Inclusion and exclusion criteria***

We included questionnaires with complete response data and excluded facilities that did not respond or those who did not perform OFC tests from January 1 to June 30, 2022.

***Statistical analyses***

Data were analyzed using SPSS version 25 (IBM Corp., Armonk, NY, USA) software. Data are expressed as n (%) or median values with 25–75th percentile interquartile ranges (IQRs). Continuous variables were analyzed using a Mann–Whitney U test. Statistical significance was set at a two-tailed p-value of <0.05.

***Sample size calculation***

This study comprised a complete survey of all facilities; therefore, the sample size was not calculated for the primary outcome. For secondary outcomes, a Mann–Whitney U test was performed to compare between two groups, and the sample size was calculated using G*Power 3.1.9.7 software (Erdfelder, Faul, & Buchner, 1996). We hypothesized that there would be a ratio of 2:1 in terms of the number of facilities with universal screening and the number of facilities without universal screening. Power was set at 0.9 and alpha was 0.05. The optimal sample size was calculated to be >55 (without a universal screening test) and 109 (with a universal screening test).

**Supplementary Table**

**Table S1.** Universal preadmission screening tests for COVID-19 for patients and their family members

|  | Patients | Family members |
| --- | --- | --- |
| Number of facilities that performed screening tests | 110 | 64 |
| Number of screening tests | 8219 | 3172 |
| Number of positive screening tests | 45 (0.5%) | 5 (0.2%) |

The positive test rate for COVID-19 was 0.5% (45/8219) for all patients and 0.2% (5/3172) for all family members.

COVID-19, coronavirus disease 2019

**Supplementary Figures**


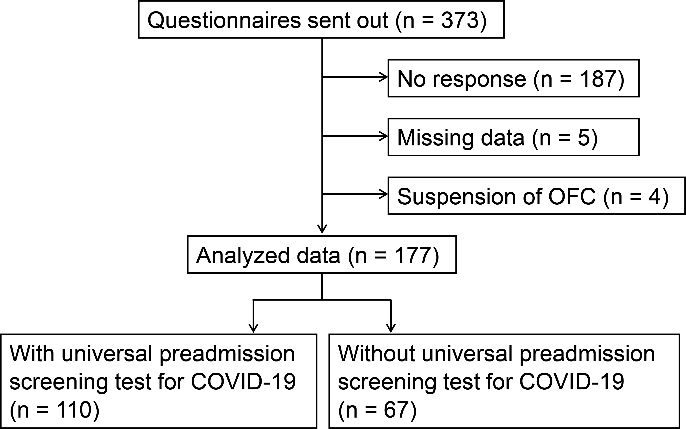


**Figure S1.** Enrollment. We invited 373 pediatric training facilities to complete an online questionnaire concerning implementation of inpatient OFC tests from January 1 to June 30, 2022. Of these, 186 facilities responded to our online questionnaire (response rate: 50%). Five were excluded due to missing data and four were excluded because they had suspended OFC tests during the COVID-19 pandemic. COVID-19, coronavirus disease 2019; OFC, oral food challenge.


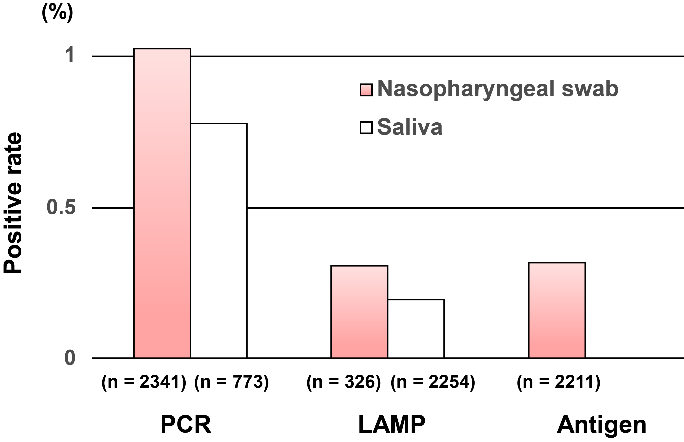


**Figure S2.** The positive COVID-19 rate among universal preadmission screening tests for patients. The positive rate for patients was the highest in those who had undergone PCR tests derived from nasopharyngeal swabs (1%, 24/2341), followed by PCR tests derived from saliva samples (0.8%, 6/773), rapid antigen tests derived from nasopharyngeal swabs (0.3%, 7/2211), LAMP tests derived from nasopharyngeal swabs (0.3%, 1/326), and LAMP tests derived from saliva samples (excluding duplication) (0.2%, 4/2254). COVID-19, coronavirus disease 2019; LAMP, loop-mediated isothermal amplification; PCR, polymerase chain reaction.
